# From study abroad to study at home: Spontaneous neuronal activity predicts depressive symptoms in overseas students during the COVID-19 pandemic

**DOI:** 10.3389/fnins.2023.1078119

**Published:** 2023-02-03

**Authors:** Tong Li, Xiaoyu Du, Xiang Zhang, Aiping Dong, Xianshun Yuan, Tianyi Yu, Ruiyuan Diao, Shuai Duan, Zijian Shen, Letian Yuan, Ximing Wang

**Affiliations:** ^1^Department of Radiology, Shandong Provincial Hospital Affiliated to Shandong First Medical University, Jinan, Shandong, China; ^2^Faculty of Medicine, Dentistry and Health Sciences, The University of Melbourne, Melbourne, VIC, Australia; ^3^College of Sports Medicine and Rehabilitation, Shandong Provincial Hospital Affiliated to Shandong First Medical University, Jinan, Shandong, China; ^4^Department of Radiology, The Second Affiliated Hospital of Shandong University of Traditional Chinese Medicine, Jinan, China

**Keywords:** COVID-19 pandemic, fMRI, overseas college students, spontaneous neuronal activity, depression

## Abstract

The objective of this study was to evaluate symptoms of depression and anxiety as well as changes in spontaneous neuronal activity in college students studying abroad during the coronavirus 2019 (COVID-19) pandemic. We examined functional brain changes using resting-state functional magnetic resonance imaging (fMRI), the amplitude of low-frequency fluctuations (ALFF), and regional homogeneity (ReHo) in overseas students with enforced isolation due to the COVID-19 pandemic. Additionally, emotional assessments were administered to determine the severity of depression and anxiety. The questionnaire results showed that anxiety and depressive symptoms differed between overseas students (i.e., those attending an overseas college virtually) and local students (i.e., those attending a local college in person). The fMRI data revealed higher ALFF values in the bilateral superior medial frontal gyrus, bilateral pre-central gyrus, left insula, and left superior temporal gyrus as well as lower ALFF values in the bilateral paracentral lobule (supplementary motor area) in overseas students. Moreover, ReHo analysis also revealed significant differences between overseas students and local students. Compared with local students, overseas students showed significantly increased ReHo in the right inferior frontal and superior temporal gyri and decreased ReHo in the bilateral paracentral lobule, bilateral superior medial frontal gyrus (supplementary motor area), and bilateral pre-central gyrus. In addition, in overseas students, altered ReHo in the cluster including the left superior and medial frontal gyri, pre-central gyrus, and paracentral lobule was significantly positively correlated with Self-Rating Depression Scale scores. Thus, spontaneous brain activity in overseas students changed during the COVID-19 pandemic. This change in brain function might be related to depression and anxiety symptoms. These results suggest that mental health services are needed to decrease the risk of anxiety and depression among college students studying abroad during the COVID-19 pandemic.

## 1. Introduction

Coronavirus disease 2019 (COVID-19) has swept across the world, leading to a global pandemic (Wang C. et al., 2020) and unprecedented changes in daily routines and social life. The impacts of this pandemic on people have been multifaceted, including short-term changes to physical, psychological, and emotional wellbeing as well as long-term changes in specific populations such as study-abroad students or older people. Students previously studying abroad had to stay in their home country because of the global lockdown initiated by various countries due to the COVID-19 pandemic, attending classes online. However, the pandemic and subsequent lockdowns imposed a variety of unprecedented challenges among study-abroad students. For example, these students had to receive online instruction in their university curricula and limit their daily activities to indoor areas (Shah et al., 2020); this lockdown stage limited interaction with peers and reduced sports activity. However, humans are inherently social, and the formation of social bonds is fundamental to survival as well as healthy cognitive, emotional, hormonal, and immune functions (Smith, 2012). Indeed, universities are one of the most important social environments. Unfortunately, during the COVID-19 pandemic, many college students have been unable to attend school, which has limited their interactions with peers, in addition to the gap imposed by social distancing measures. Lee et al. found that school routines played a positive role in coping specifically for young people with mental health issues (Lee, 2020). Moreover, social touch is essential for the healthy development of cognitive function, emotions, attachments, and relationships (Cascio et al., 2019). Wang et al. reported that periods without school are associated with increased screen time, irregular sleep patterns, and decreased physical activity (Wang G. et al., 2020). Therefore, school life is important for college students.

Students, especially overseas students, experienced enforced isolation during the lockdowns; their mental health might have been altered due to the COVID-19 pandemic. Recent studies have also indicated possible short-term effects of exposure to COVID-related stressors at this stage of life, including distress and hopelessness as well as irregular food intake, and possible long-term consequences, including altered brain circuitry, lack of emotional processing, psychiatric disorders, and suicidal thoughts (de Figueiredo et al., 2021). Evidence has suggested that the COVID-19 pandemic has generally increased levels of stress and depression among college students (Lee, 2020); moreover, the proportion of individuals reporting depression, anxiety, and/or suicidal thoughts is alarming (Wang G. et al., 2020; Wang C. et al., 2020; de Figueiredo et al., 2021). An online questionnaire revealed that pandemic-related factors might be associated with a higher risk of depressive symptoms among college students in China (Cascio et al., 2019). Therefore, the current study focused on the impact of the COVID-19 pandemic on the mental health of overseas college students, especially students who planned to continue studying abroad but were prevented by the closure of educational activities at universities in other countries.

Structural alteration of the brain is usually considered a long-term result of neural plasticity. Resting-state functional magnetic resonance imaging (rs-fMRI) is generally recognized as a non-invasive neuroimaging technique and is widely utilized to investigate resting-state brain activity *via* blood oxygen level-dependent signals (Fox and Raichle, 2007). Hence, resting-state brain activity serves as a baseline, reflecting spontaneous neural activity, and rs-fMRI data provide a more feasible method of assessing the brain’s functional response to instantaneous extrinsic behaviors. The amplitude of low-frequency fluctuations (ALFF) and regional homogeneity (ReHo) are common measures used to describe regional properties of brain activity in rs-fMRI studies (Zang et al., 2004; Yu-Feng et al., 2007). ALFF values are used to indicate the intensity of neural activity at the single-voxel level (Yu-Feng et al., 2007), while ReHo values are used to characterize the synchronization of fluctuations of a voxel with its neighboring voxels (Zang et al., 2004). These two metrics are two data-driven approaches widely established as having high reliability. ReHo may be more sensitive to regional changes than ALFF. However, ALFF and ReHo complement each other in terms of detecting global spontaneous activity (An et al., 2013). Therefore, the ALFF and ReHo methods might be jointly applied to collect more data in the field of cognitive neuroscience.

Here, we identified differences in spontaneous whole-brain activity between college students who planned to continue to study abroad but were prevented by the closure of educational activities in foreign universities (overseas students) and those who did not originally plan to study abroad and attended school in China (local students). We collected rs-fMRI data from these two groups to analyze ALFF and ReHo values. Furthermore, the relationship between brain regions with significant differences in these values and behavioral data was analyzed. We specifically hypothesized that the activity of neural circuits would be differentially altered in the two groups. We hypothesized that college students may have experienced not only short-term but also long-term consequences of COVID-19 stressors. In the context of COVID-19 lockdowns, we predicted that the stress of social isolation and other pandemic-related adversities affected the brains of these students. However, the precise changes in the brains of college students, especially in key regions associated with emotion regulation, empathy, and decision-making skills, remain unclear.

## 2. Materials and methods

### 2.1. Subjects

**FIGURE 1:**
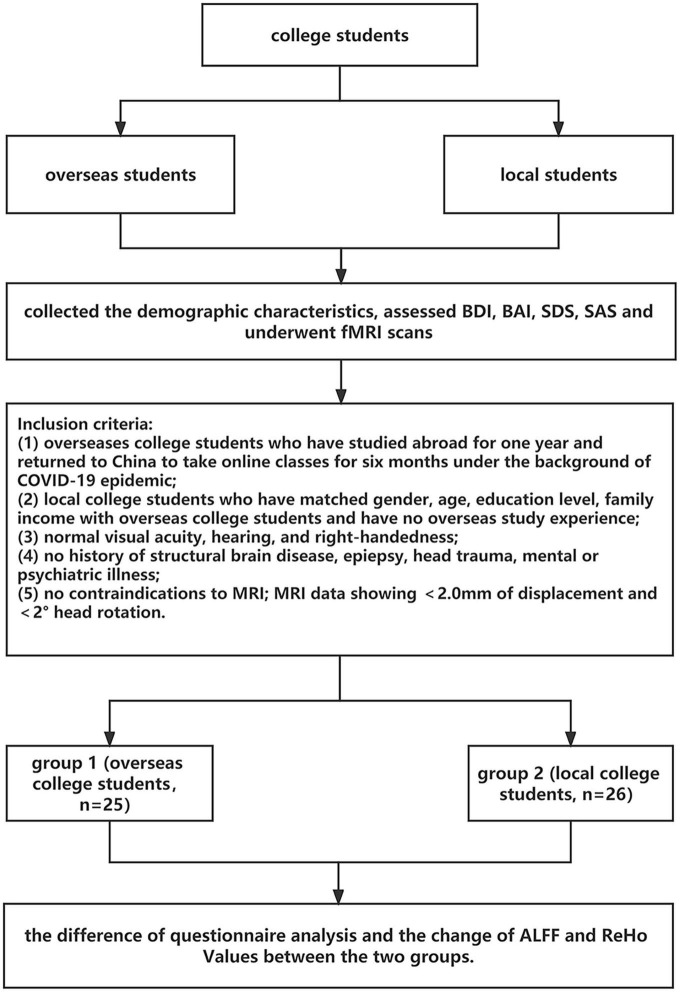
Flowchart of participant recruitment and data collection.

We recruited 51 subjects by posting physical and digital (social media) advertizements. One group was comprised of 25 overseas students who had studied abroad for over 1 year (age, mean ± standard deviation: 22.68 ± 3.38 years). If there was no COVID-19 pandemic, they would have continued to study abroad. However, because international flights were halted to prevent imported cases of COVID-19, these students had to stay in China and take online classes. In addition, we recruited 26 local college students (age, mean ± standard deviation: 21.69 ± 1.78 years) attending universities in China, matched by age and sex. No subjects had a history of neurological, medical, or psychiatric conditions or a history of severe head trauma according to screening; all subjects were screened for MRI contraindications after providing written consent to participate. The Chinese Hand Preference Questionnaire, adapted from the Edinburgh Handedness Inventory, was used to screen participants for handedness; only right-handed individuals were included. Clinical and demographic data for the two groups are shown in Table 1. Figure 1 shows the participant recruitment and data collection procedures.

**TABLE 1:** Demographic and questionnaire data of college students in the overseas and local groups.

Dimensions	Overseas	Local	*T* value	*P* value (two-tailed)
*N*	*n* = 25	*n* = 26	–	–
Age	22.47 ± 3.38	21.69 ± 1.78	1.034	0.306
Education (years)	14.12 ± 1.48	14.73 ± 0.53	1.89	0.069
Parental companionship (hours)	5.10 ± 6.89	7.44 ± 7.54	**−**1.13	0.264
Sports activity (hours)	1.07 ± 1.33	1.00 ± 0.69	0.224	0.824
Online classes (hours)	2.83 ± 1.13	4.08 ± 0.97	**−**4.194	**0.001**
Sleep (hours)	7.67 ± 1.42	7.74 ± 0.69	**−**0.231	0.818
BDI score	7.13 ± 5.17	1.62 ± 2.16	4.988	**0.001**
SDS score	0.22 ± 0.12	0.16 ± 0.10	1.927	**0.030**
BAI score	30.63 ± 9.65	26.08 ± 4.26	2.186	**0.017**
SAS score	33.29 ± 7.01	29.81 ± 4.98	2.038	**0.023**

Data are presented as the mean ± standard deviation (SD). Comparisons were calculated using independent sample t tests, and the significance threshold was set at *P* < 0.05. BDI, Beck Depression Inventory; BAI, Beck Anxiety Inventory; SDS, Self-Rating Depression Scale; SAS, Self-Rating Anxiety Scale. Bold values indicate the *P* < 0.05.

All procedures were approved by the Ethics Committee of Shandong Provincial Hospital Affiliated to Shandong First Medical University. Each volunteer signed an informed consent form, which was approved by the committee. All methods in our study were carried out in accordance with the principles outlined in the Declaration of Helsinki.

### 2.2. Questionnaire analysis

The Beck Depression Inventory (BDI) (Beck et al., 1988b) was originally developed by Beck et al. (1961). This questionnaire was designed to assess the severity of depressive symptoms. Participants rate each item on a four-point scale. Evidence indicates that the reliability and validity of the Chinese version of the BDI scale are acceptable (Sun et al., 2017). The Beck Anxiety Inventory (BAI) (Beck et al., 1988a) is a scale measuring the severity of self-reported anxiety symptoms. Participants are asked to rate symptoms of anxiety (e.g., “fear of losing control” and “racing or pounding heart”) using a four-point scale. The Chinese version of the BAI was demonstrated to provide satisfactory reliability and convergent validity on psychometric indices (Liang et al., 2018). In addition, Zung’s Self-Rating Depression Scale (SDS) (Zung, 1965) and Self-Rating Anxiety Scale (SAS) (Zung, 1971) are two widely used self-report scales that assess depression and anxiety. The two scales cover both affective and somatic symptoms. Participants are asked to answer the questions based on their experiences and feelings using a four-point Likert scale ranging from 1 to 4. The SAS and SDS have good internal consistency and can be used to measure depression and anxiety. Moreover, previous studies have used the SAS (Wang Z. H. et al., 2020), SDS (Tong Li et al., 2021), BAI and BDI (Ganji et al., 2022) to assess the impact of the COVID-19 pandemic on the mental health of college students. Hence, in the current study, all participants completed these scales to assess the severity of symptoms of depression and anxiety. For all measures, descriptive statistics were performed. Subsequently, we conducted independent-sample *t* tests to compare overseas students and local students. Significant results were reported at *P* < 0.05.

### 2.3. MRI image acquisition

Magnetic resonance imaging scans were performed on a Siemens 3.0 T Prisma MR system at the Shandong Provincial Hospital Affiliated to Shandong First Medical University (Jinan, Shandong, China). We used a 64-channel head coil for whole-brain scanning. Anatomical images were collected using a high-resolution T1-weighted 3-dimensional magnetization-prepared rapid-acquisition gradient-echo pulse sequence with the following acquisition parameters: repetition time (TR) = 2,530 ms, echo time (TE) = 2.98 ms, inversion time (TI) = 1,100 ms, field of view (FOV) = 256 mm, acquisition matrix = 256 × 256 mm^2^, flip angle = 7°, and slice number = 192 slices. During the rs-fMRI scan, a T2*-weighted gradient-echo echo-planar-imaging (GRE-EPI) sequence, which is sensitive to blood oxygen level-dependent (BOLD) signals, with the following parameters was utilized: TR = 2,000 ms, TE = 30 ms, FOV = 220 × 220 mm^2^, matrix size = 64 × 64, slice thickness = 3.5 mm, flip angle = 90°, slices = 33, transverse orientation, and 25% distance factor. A total of 210 whole-brain volumes were acquired. All participants were instructed to relax with their eyes closed and not think of anything in particular during the entire scan session.

### 2.4. MRI data analysis

#### 2.4.1. Data pre-processing

MRI data were analyzed using a toolbox for Data Processing and Analysis for Brain Imaging (DPABI^1^) (Yan et al., 2016) and Statistical Parametric Mapping software (SPM12^2^) based on MATLAB 2021a. Data analysis included pre-processing and statistical analysis. Pre-processing involved the following steps. First, the DICOM MRI data format was converted to NIFTI images. The initial 10 time points were discarded to allow for steady-state magnetization, and the remaining volumes underwent slice-timing correction and spatial realignment to correct for head motion using a six-parameter (rigid body) linear transformation; participants with head movement > 2 mm or head rotation > 2° were excluded. Next, the mean functional images were coregistered to the high-resolution T1-weighted images, and the high-resolution T1-weighted images were segmented into gray matter, white matter and cerebrospinal fluid (Ashburner and Friston, 2005). These individual images were spatially normalized to the Montreal Neurological Institute template with diffeomorphic anatomical registration through the exponentiated lie algebra (DARTEL) (Ashburner, 2007) tool (resolution: 3 mm × 3 mm × 3 mm). This step was followed by regressing nuisance variables, including the time series of six head motion parameters, the white matter signal and the cerebral spinal fluid signal, using a general linear model.

#### 2.4.2. ALFF analysis

ALFF analysis was performed with DPABI (Yan et al., 2016). For ALFF values, the generated images were processed using spatial smoothing with a 6 mm FWHM Gaussian kernel. Then, we transformed each voxel of the filtered time series into the frequency domain with a fast Fourier transform, and the power spectrum was calculated. After calculating the square root of the signal across 0.01–0.1 Hz for each voxel, subtracting the average value, and dividing by the whole-brain voxel deviation, the ALFF values for all participants were converted to an m-distribution to achieve standardization. Finally, standardized whole-brain ALFF maps were obtained for each participant (Zang et al., 2007).

#### 2.4.3. ReHo analysis

After a 0.01–0.1 Hz bandpass filter was applied, the unsmoothed pre-processed data were used for ReHo calculations. Kendall’s coefficient of concordance (KCC) was applied to calculate the synchronicity of the time series of the fMRI signal of this voxel and those of its adjacent 26 voxels. Each individual ReHo map was generated by calculating the KCC using a voxel wise method. Each individual’s ReHo map was converted into a standard *z* score map by subtracting the global mean and dividing by the standard deviation within the whole-brain ReHo map. Then, a 6 mm FWHM Gaussian kernel was used to complete the smoothing process to reduce noise. Finally, the ReHo value of each participant was divided by the average ReHo value of all participants in each group.

#### 2.4.4. Statistical analysis

After pre-processing, statistical analysis was performed using SPM12. The two-sample *t* test was used to determine brain regions that significantly differed in ALFF and ReHo. Note that age, sex, education level, mean frame displacement (FD), and physical activity were regressed in the between-group comparison. An initial threshold of *P* < 0.001 (uncorrected) was applied, and results that survived family wise error (FWE) correction at a cluster-level threshold of *P* < 0.05 were reported.

To investigate the relationship of differences in ALFF and ReHo values with behavioral scores, partial correlation analysis was performed using SPSS 24.0 (SPSS, Inc., Chicago, IL, USA). Age, years of education, sleep duration, duration of online classes, and duration of sports activity were regressed out. First, the mean ALFF and ReHo values of brain regions with significant differences were individually extracted. Then, partial correlation analyses were applied to examine possible associations of these ALFF and ReHo values with behavioral scores. The statistical threshold was set at *P* < 0.05 using a Benjamini-Hochberg false discovery rate (FDR) correction with MATLAB to explore the most significant correlations.

## 3. Results

### 3.1. Demographic and questionnaire data

The demographic characteristics of overseas students and local students are listed in Table 1, including the means and standard deviation for age, years of education, duration of parental accompaniment, duration of physical activity, duration of online classes, duration of sleep and scores on each questionnaire. As shown in Table 1, significant group differences were found in the online class duration, BDI score, SDS score, BAI score, and SAS score (*P* < 0.05). Compared with local students, overseas students had significantly higher BDI, SDS, and BAI scores.

### 3.2. ALFF and ReHo results

Differences in ALFF and ReHo values (FWE corrected *P* < 0.05) between overseas students and local students are shown in Table 2 and Figure 1. The following brain regions had significantly higher ALFF values in overseas students than in local students: the bilateral superior frontal gyrus, bilateral superior temporal gyrus, bilateral inferior frontal gyrus, left insula, right superior frontal gyrus, and right medial frontal gyrus. In contrast, the ALFF values of the bilateral paracentral lobule (in the supplementary motor area), bilateral pre-central gyrus, bilateral post-central gyrus, and right cerebellum posterior lobe were lower in overseas students.

**TABLE 2:** Brain regions with significant alterations in ALFF and ReHo values between overseas students and local students.

Group	*N*	Regions	BA	Voxels	*X*	*Y*	*Z*	*T*	*P*_*FWE*_
**ALFF**									
	1	Left insula Left superior temporal gyrus	13	70	−42	9	−9	5.70	0.011
Overseas > Local (increases)	2	Left inferior frontal gyrus Left superior temporal gyrus	45/47	126	−57	18	−9	5.45	0.001
	3	Right medial frontal gyrus Right superior frontal gyrus	10	60	6	63	15	5.15	0.024
	4	Right superior temporal gyrus Right inferior frontal gyrus	38/47	62	57	24	−9	4.05	0.020
Overseas < Local (decreases)	1	Left and right medial frontal gyrus Left and right paracentral lobule Left and right pre-central gyrus Left and right post-central gyrus (supplementary motor area)	6/4	158	0	−21	69	4.94	0.001
	2	Right cerebellum posterior lobe		92	42	−78	−54	4.13	0.002
**ReHo**									
Overseas < Local (increases)	1	Right inferior frontal gyrus Right superior temporal gyrus	47	124	57	30	−24	5.59	0.001
Overseas > Local (decreases)	1	Left pre-central gyrus Left middle frontal gyrus Left paracentral lobule Left medial frontal gyrus (supplementary motor area) Left superior frontal gyrus	4/3/6	111	−12	−12	69	6.06	0.001
	2	Right pre-central gyrus Right medial frontal gyrus (supplementary motor area) Right superior frontal gyrus	6	121	27	−21	69	4.76	0.001

This table indicates the changes in ALFF and ReHo for the two groups. X, Y, Z = MNI coordinates. The results reported are significant at the peak level with *P* < 0.001 (uncorrected) and at the cluster level with *P* < 0.05 (FWE-corrected).

**FIGURE 2:**
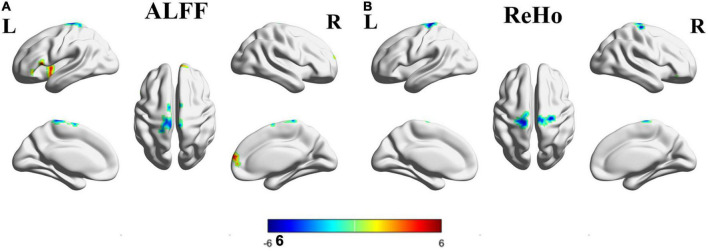
Brain regions with significantly different ALFF and ReHo values between the overseas group and the local group. Colors (blue-red) represent decreased and increased ALFF or ReHo values. L, left; R, right; **(A)** ALFF; **(B)** ReHo.

Compared to local students, overseas students showed significantly lower ReHo values in the bilateral frontal lobules, including the superior frontal gyrus, medial frontal gyrus (in the supplementary motor area), pre-central gyrus, paracentral lobule, and middle frontal gyrus. However, overseas students showed significantly higher ReHo values in right inferior frontal gyrus and superior temporal gyrus (Figure 2 and Table 2). In summary, compared to local students, overseas students had significantly altered ALFF and ReHo values around the frontal pole. The surviving clusters represent a significant difference at a peak level of *P* < 0.001 and cluster level of *P* < 0.05 with FWE corrections.

### 3.3. Correlations of MRI data and questionnaire data

**FIGURE 3:**
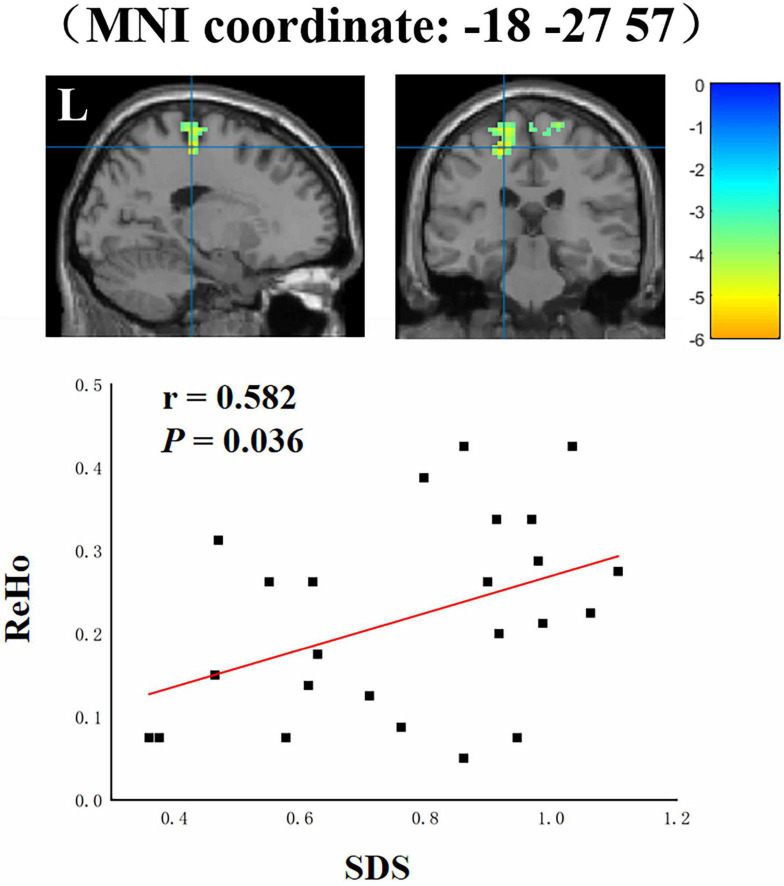
Correlations between ReHo values and SDS scores in overseas students. L, left.

Partial correlation analysis was applied to investigate correlations of anxiety and depressive symptoms with spontaneous brain activity. In overseas students, the decreased ReHo values in the cluster (*x* = −18, y = −27, *z* = 57, *r* = 0.582, *P* = 0.036) including the left superior and medial frontal gyri, pre-central gyrus, and paracentral lobule were significantly positively correlated with SDS scores (Figure 3). However, in local students, no significant relationships were found between ALFF or ReHo values and depression or anxiety scores.

## 4. Discussion

To the best of our knowledge, this is the first rs-fMRI study to investigate differences in spontaneous brain activity between overseas students and local students against the background of the COVID-19 pandemic. Our main findings are as follows. (a) Compared with local students, overseas students had altered ALFF and ReHo values in the following regions: the bilateral frontal lobule, including the medial frontal gyrus, middle frontal gyrus, superior frontal gyrus, pre-central gyrus, and paracentral lobule. (b) In overseas students, the altered ReHo values in the cluster including the left superior and medial frontal gyri, pre-central gyrus, and paracentral lobule were significantly positively correlated with SDS scores.

Compared to local students, overseas students had notably altered ALFF and ReHo values in the bilateral frontal lobule. Abnormalities in this region, which includes the prefrontal cortex (Ye et al., 2012), middle frontal gyrus (Zhang et al., 2011), and insula (Avery et al., 2014), have been frequently reported in fMRI studies of major depressive disorder. Previous studies have indicated that the middle frontal gyrus is involved in regulating the strength of reactions to emotional stimuli and that the superior frontal gyrus is a core brain region related to emotion regulation processes, especially regarding feelings of amusement (Frank et al., 2014; Zhang et al., 2021). According to these findings, alterations to these regions might influence the development of depressive symptoms. In addition, these regions are crucial areas of the dorsolateral prefrontal cortex, which has been closely linked to depression. For example, Kaiser et al. suggested that higher functional activity in the dorsolateral prefrontal cortex is frequently observed in major depressive disorder (Kaiser et al., 2015). Furthermore, the prefrontal cortex is a critical region for attention regulation and emotional judgment. The prefrontal cortex is the primary region targeted by repetitive transcranial magnetic stimulation to treat young patients with treatment-resistant depression (Zheng et al., 2010). These results demonstrate that dysfunction in the prefrontal cortex might appear during the early stage of depression. In the current study, we found that the superior and medial frontal gyri and pre-central gyrus (including the inferior frontal junction) displayed altered spontaneous neural activity in overseas students during the COVID-19 pandemic, suggesting abnormal brain function in these areas. We compared these results to those of previous studies on depression or anxiety and found that similar brain changes as those in individuals with depression or anxiety occurred in overseas students. Thus, the COVID-19 pandemic potentially imposed additional stressors on college students during this challenging time, leading to depression or anxiety. These findings can enhance the understanding of regions initially impaired in anxiety disorders and depression. The altered ALFF and ReHo values further suggest dysfunction in emotion-related regions in overseas students; functional impairments in these regions might directly influence the inappropriate responses to emotional events observed in overseas students.

In the current study, anxiety and depression scores were significantly higher in overseas students than in local students. These scales measure symptoms of depression and anxiety. The group differences in questionnaire scores suggest that overseas students might have a higher prevalence of anxiety and depressive symptoms than local students. These results suggest that overseas students tended to experience depression due to the COVID-19 pandemic. This finding is consistent with studies that also found poor mental health among college students during the COVID-19 pandemic (Son et al., 2020; Copeland et al., 2021).

In addition, we also found a significant positive correlation between depression severity and alterations in activity in the cluster including the left superior and medial frontal gyri, pre-central gyrus, and paracentral lobule. These regions are associated with emotion regulation (Rive et al., 2013). Increased spontaneous neural activity in these regions and a positive correlation between this activity and depression severity were found in overseas students compared to local students. These results further imply that dysfunction of the prefrontal-cortical circuit is a key factor that leads to depression.

Although our research revealed that spontaneous brain activity in overseas students changed during the COVID-19 pandemic, this study had several limitations. First, the sample size was relatively small, and more subjects and observations are needed to verify the current results. Second, this study had a cross-sectional design. In future studies, we will verify these changes using a longitudinal design. Third, future experiments should control for confounding factors (e.g., college student majors) to confirm that differences in spontaneous brain activity between overseas students and local students are linked to COVID-19 pandemic.

In summary, we observed altered patterns of brain activity in overseas students according to rs-fMRI data, with increased ALFF mainly in the PFC and decreased ALFF in the paracentral lobule. The vulnerability of overseas students to depression and anxiety is potentially related to a concerted mechanism that involves distinct regional alterations in resting-state activity in the mPFC and dlPFC. Our findings of abnormal resting-state activity in a unique frontoposterior pattern may enhance the current understanding of the neurobiological underpinnings of mental disorders in overseas students during the COVID-19 pandemic. Overall, this study of local spontaneous brain activity provided evidence of differences between overseas students and local students. In future work, we will use connectivity measures to explore the interactions among brain regions.

## Data availability statement

The original contributions presented in this study are included in the article/supplementary material, further inquiries can be directed to the corresponding author.

## Ethics statement

The studies involving human participants were reviewed and approved by Shandong Provincial Hospital Affiliated to Shandong First Medical University. The patients/participants provided their written informed consent to participate in this study.

## Author contributions

TL, XD, and XW contributed to the study conception, design, material preparation, data analysis, and writing draft. XD and XZ contributed to revising and writing manuscript. AD, XY, TY, RD, SD, ZS, and LY were performed data collection. All authors read and approved the final manuscript.

## Footnotes

1
http://rfmri.org/dpabi

2
http://www.fil.ion.ucl.ac.uk/spm/software/spm12
